# Preparation and Hydration Properties of Sodium Silicate-Activated Municipal Solid Waste Incineration Bottom Ash Composite Ground-Granulated Blast Furnace Slag Cementitious Materials

**DOI:** 10.3390/ma17102406

**Published:** 2024-05-17

**Authors:** Juan Deng, Guoxiong Wu, Yuchao Xia, Li Liu

**Affiliations:** 1 School of Civil Engineering, Chongqing Jiaotong University, Chongqing 400074, China; juandeng@mails.cqjtu.edu.cn; 2 Department of Transportation and Municipal Engineering, Chongqing Jianzhu College, Chongqing 400072, China; 3 School of He Hai, Chongqing Jiaotong University, Chongqing 400074, China

**Keywords:** MSWIBA, sodium silicate-activated, hydration properties, hydration process

## Abstract

The production of municipal solid waste incineration bottom ash (MSWIBA) is substantial and has the potential to replace cement, despite challenges such as complex composition, uneven particle size distribution, and low reactivity. This paper employs sodium silicate activation of MSWIBA composite Ground-granulated Blast Furnace slag (GGBS) to improve the reactivity in preparing composite cementitious materials. It explores the hydration performance of the composite cementitious materials using isothermal calorimetric analysis, Fourier-transform infrared (FTIR) spectroscopy, XRD physical diffraction analysis, and SEM tests. SEM tests were used to explore the hydration properties of the composite gelling. The results show that with an increase in MSWIBA doping, the porosity between the materials increased, the degree of hydration decreased, and the compressive strength decreased. When the sodium silicate concentration increased from 25% to 35%, excessive alkaline material occurred, impacting the alkaline effect. This inhibited particle hydration, leading to a decrease in the degree of hydration and, consequently, the compressive strength. The exothermic process of hydration can be divided into five main stages; quartz and calcite did not fully participate in the hydration reaction, while aluminum did. The vibrational peaks of Si-O-Ti (T = Si and Al) were present in the material. The vibrational peaks of XRD, FTIR, and SEM all indicate the presence of alumosilicate network structures in the hydration products, mainly N-A-S-H and C-A-S-H gels.

## 1. Introduction

Solid waste production is enormous and many countries face serious issues with municipal solid waste (MSW) and environmental pollution. Selecting high-temperature incineration to treat MSW is the most effective way to reduce volume [1,2]. In general, MSW incineration treatment can reduce its mass by 70% and its volume by 90% [2,3], while the rate of unburned ash in the ash residue is 0.7–2%. The residual rates of MSWIBA and fly ash generation from MSW incineration are approximately 20% and 2% [4], respectively. Even after incineration, a large amount of MSW incineration bottom ash (MSWIBA) is still generated [5].

The rapid development of modern society requires alternative materials for sustainable development [6]. Provis pointed out that alkali-activated materials offer real possibilities to improve the sustainability of civil construction [7]. Ordinary Portland cement (OPC) consumes a considerable amount of non-renewable resources, as its production process accounts for approximately 8–10% of anthropogenic CO_2_ emissions [8,9,10]. When using reactive alkali materials in concrete production, the CO_2_ emissions are approximately 3.6 times lower than in conventional concrete production [11]. Several studies have shown that MSWIBA is potentially chemically active and can be blended with GGBS as a potential replacement material for cement through the action of alkali exciters [12].

MSWIBA is characterized by an unstable source, complex composition, non-uniform particle size distribution, and low reactivity, so it is necessary to adopt an activation treatment method to address the difficulties and problems of MSWIBA in engineering utilization [13]. Numerous scholars have explored the effect of MSWIBA at different blending levels on cement mortar or cement and found that bottom slag reduces the slump value [5,13,14,15,16,17,18,19,20,21]. In contrast, the opposite trend was observed when used as a cement substitute. The 28-day compressive strength of the latter was similar to or slightly higher than that of the control mixture (without the addition of MSWIBA) [22,23]. Lin et al. prepared cubic specimens to test the compressive strength of hardened slurries of cementitious materials made from slag and cement by melting MSWIBA into the slag and grinding it into powder. The results showed that the 28- and 90-day compressive strengths of the specimens were 84~90% and 95~110% of ordinary cement specimens, respectively [22,24,25]. The effect of Al_2_O_3_ on MSWIBA, by melting the mixture into slag and pulverizing it to replace part of the cement, has some merit [22,26].

Alkali excitation technology uses alkali exciters to activate the potential activity in MSWIBA and convert it into a material with gelling properties [27]. MSWIBA includes a large number of active components, such as silicon and aluminum, which have the potential to be alkali-activated [25]. The material undergoes hydration during the alkali activation process, the most critical chemical reaction being that between the aluminosilicate powder and the alkali exciter to produce a hardened slurry with high strength [25,27]. According to P. Kryvenko [28], the resulting geopolymer network consists of SiO_4_ and AlO_4_ tetrahedra, both with oxygen atoms attached, and the negative charges are balanced by positive ions (e.g., K^+^, Na^+^, Ca^2+^, and Li^+^) in the cavity framework [27,29,30]. The secondary binder products formed during alkali activation (including layered double hydroxides in the alkali-activated slag) are essential in determining the final properties of the material [7].

MSWIBA has a relatively stable (crystalline) chemical structure and low reactivity when in contact with water; when used as a cementitious material, it needs to be activated to improve its properties. The most common activation treatments for this purpose are physical milling activation treatment and activation treatment using alkaline substances [17,31].

The most common alkali activators are sodium hydroxide (NaOH) and sodium silicate (Na_2_SiO_3_, water glass) [32,33]. It is suggested that for alkali-activated slag cementitious materials, sodium silicate should be used [34,35], taking into consideration factors such as the molar concentration, the solution–binder ratio (s/b), and alkalinity [32,36,37].

In this paper, we used sodium silicate as an alkali activator and considered different ratios of Na_2_SiO_3_, NaOH, and MSWIBA to prepare alkali-activated materials. We used isothermal calorimetric analysis, Fourier-transform infrared (FTIR) spectroscopy, XRD physical phase diffraction analysis, and SEM, among other tests, to explore the hydration properties of composite cementation research. This aimed to promote the harmless disposal of MSWIBA and waste reuse, reduce the amount of cement, achieve energy saving and emission reduction, and promote the comprehensive reuse of the MSWIBA of this solid waste. These efforts provide theoretical references for the treatment of and application of MSWIBA.

## 2. Methods

### 2.1. Experimental Materials

#### 2.1.1. MSWIBA and GGBS

The MSWIBA was generated by the waste incineration company Chongqing Tongxing in China Chongqing using the German Martin SITY2000 reverse thrust tilting grate technology to burn the MSW from the Chongqing area and obtain the residue. After incineration, the ferrous metal was washed, magnetically selected, and then crushed by a crusher after natural drying in the stockpile plant.

The MSWIBA was processed in a muffle furnace at a high temperature of 1000 °C for three hours and then ground by a planetary ball mill for 30 min to improve its reactivity.

By weighing 2~3 g of pre-treated MSWIBA degassed in a vacuum environment at 110 °C for 16 h using a fully automated specific surface and pore size analyzer (Quantachrome Instruments, Inc. nova-4000, Boynton Beach, FL, USA) for testing and by putting MSWIBA under an analytical temperature of −196 °C and a relative pressure interval of P/P0 = 0.01~0.995, low-pressure N2 adsorption/desorption isotherms of MSWIBA after incineration were obtained.

**Figure 1:**
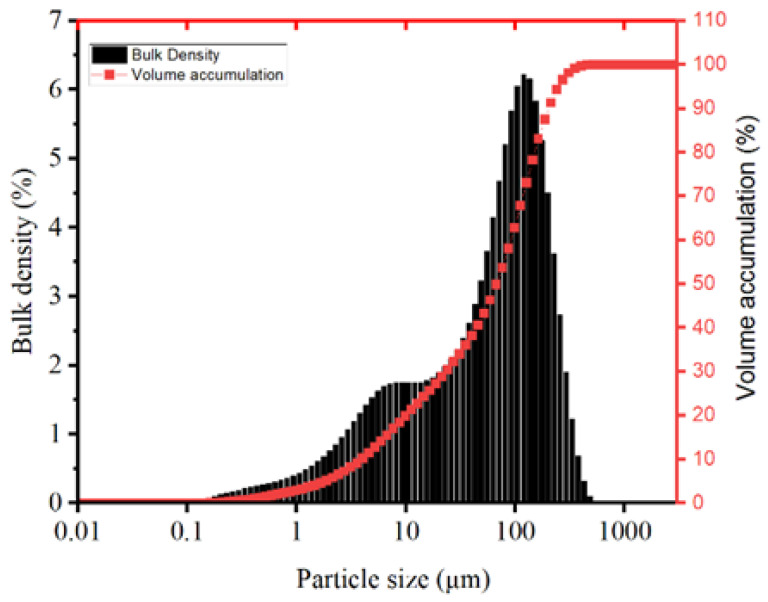
MSWIBA particle size distribution.

The treated MSWIBA was tested using the BET method to obtain a specific surface area of 1.705 m^2^g^−1^ and an average pore size of 3.388 nm. The particle size distribution of the period analyzed by the laser particle sizing instrument is shown in Figure 1.

Using an X-ray fluorescence spectrometer (Rigaku Corporation, Co. ZSX Primus III+, Tokyo, Japan) on the MSWIBA and S95 GGBS, the chemical composition of the MSWIBA was determined, as shown in Table 1. According to the National Standards of China definition of the Ground-granulated Blast Furnace slag (GGBS) for Cement [38], the alkalinity index of the MSWIBA, expressed as the alkalinity index (Mo = (CaO + MgO)/(SiO_2_ + AL_2_O_3_)), was approximately 0.68, while an Mo < 1 indicates acidic GGBS.

In this paper, S95 Ground-granulated Blast Furnace slag (GGBS) was selected and its density was obtained through experimental measurements, with a loss on burning of 0.84% and a water content of 0.45%. The 7-day activity index was 84.20% and Mo = (CaO + MgO)/(SiO_2_ + AL_2_O_3_) = 1.08, indicating alkalinity according to the National Standards of China [39].

#### 2.1.2. Sodium Silicate

Liquid sodium silicate, also known as water glass, was used in this paper as an AR analytically pure product with a modulus of 2.25, a Baume degree of 43.5, a density of 1.51 g/mL, a Na_2_O content of 13.75 w%, a silica SiO_2_ content of 29.99 w%, and a transparency ≥85 w%. It was produced by China Jiashan County Yourui Refractories Co. China, Jiaxing, to which 2 w% NaOH white homogeneous granular solid (Xilong Science Co. China, Chongqing) was added.

#### 2.1.3. Other Materials

ISO reference sand was used for the tests, composed of natural round siliceous sand with a SiO_2_ content of no less than 98%, meeting the requirements of National Standards of China [40]. Except for the isothermal calorimetry experiment, which used deionized water, the other experiments used tap water.

### 2.2. Methods

#### 2.2.1. Material Mixing

According to the results of previous experiments, five groups of mixing ratios were selected for comparative analysis, as shown in Table 2. According to the total amounts of MSWIBA, GGBS, and sodium silicate alkali components, along with the amount of water added, the water–cement ratio (W/B) was fixed at 0.50 for the preparation of the slurry. The MSWIBA content was the lowest in the B5S25 experimental group, while the B29S25, B29S15, and B29S35 experimental groups had the highest contents, followed by B17S25. The sodium silicate content was the highest in the B29S35 experimental group, followed by B5S25, while B29S15 had the lowest content.

#### 2.2.2. Experimental Methods

1. Compressive strength

According to the Test Procedure for Cement and Concrete in Highway Engineering and Test Method for Strength of Cementitious Sand (ISO Method) [40], the compressive and flexural strengths of cementitious sand are determined by molding. The size of the steel–plastic specimen was in accordance with the requirements of JC/T726, with the inner length of the test specimen being 40 × 40 × 160 mm. The specimen and the test specimen were cured in a standard curing room for 20 to 23 h. After 20 to 23 h of curing, the specimen was demolded with a machine. After demolding, the compressive and flexural strengths of the specimens were tested according to the curing requirements until the specified age. According to the molding specification, after being maintained in a standard maintenance room for 3 and 28 days, the different alkali excitation ratios of the MSWIBA composite cementitious cemented sand specimens were measured.

2. Isothermal calorimetry experiment

The heat of hydration of the composite slurry was determined using a TAM Air isothermal calorimeter produced by TA Instruments, Inc. (New Castle, DE, USA). The test time was 7 days, the calorimeter temperature was 20 °C, the water–cement ratio (W/B) was 0.5, and the material composition was consistent with Table 2. Before the experiment, the slurry was thoroughly mixed and poured into the isothermal calorimeter. After exothermic stabilization (30 min), the experimental results were recorded.

3. Hydration product analysis

The net slurry was prepared according to the Chinese national standard (GB/T1346-2011). All of the powdered materials (i.e., the GGBS and alkali components) were mixed, and then water was continuously stirred in until a homogeneous slurry was formed. The AASC slurry was hydrated at 20 ± 2 °C and 95 ± 5 RH for 3, 7, and 28 days. The AASC samples were then pulverized into small pieces and soaked in anhydrous ethanol for three days to stop hydration.

The samples were then dried in an oven at 55 °C for 48 h, crushed to make small pieces, and then vacuum processed. The microscopic morphology of the different materials was observed using a scanning electron microscope. Elemental analysis was conducted using a scanning electron microscope and an energy spectrometer. Some of the small pieces were crushed and milled for 15 to 20 min and passed through a 200 mesh square hole sieve to carry out the XRD, SEM, and FTIR spectroscopy tests.

This paper used an X-ray diffractometer (Bruker, D8Advance, Karlsruhe, Germany) to analyze the physical phase, set-hardened net paste, and mortar of the raw materials. The operating parameters of the instrument were Cu target, Kα rays, λ = 0.015 nm, X-ray generation power = 3 kw, and step size = 0.02°. The retrieved physical phases were analyzed using Jade 6.5 and HighScore Plus (3.0.5) for mineral structure analysis software. The 2023 database in X′Pert HighScore software (3.0.5) was compared to determine the crystalline phase of the material by analyzing the different peaks in the XRD pattern and comparing them with known crystal structures [41].

The molecular structure and vibrational modes of the composite cementitious materials were tested using an FTIR spectrometer utilizing a diffuse reflectance technique, which permits the incoming beam to be reflected from the ground sample to the overhead mirror, where diffusely scattered light is collected and measured in the detector.

The surface morphology of the cementite samples was observed via field emission group scanning electron microscopy (SEM) using a scanning electron microscope (Carl Zeiss AG Co. ZEISS supra55, Germany, Oberkochen), and the samples were prepared in the form of blocks with a surface area of approximately 0.5 cm^2^ and a thickness of approximately 1 mm. One side of the block was polished with sandpaper to facilitate bonding on the sample stage, and the other was left untreated for observation.

## 3. Results and Discussion

### 3.1. Mechanical Properties

#### 3.1.1. Mechanical Properties of Different MSWIBA Doping Levels

**Figure 2:**
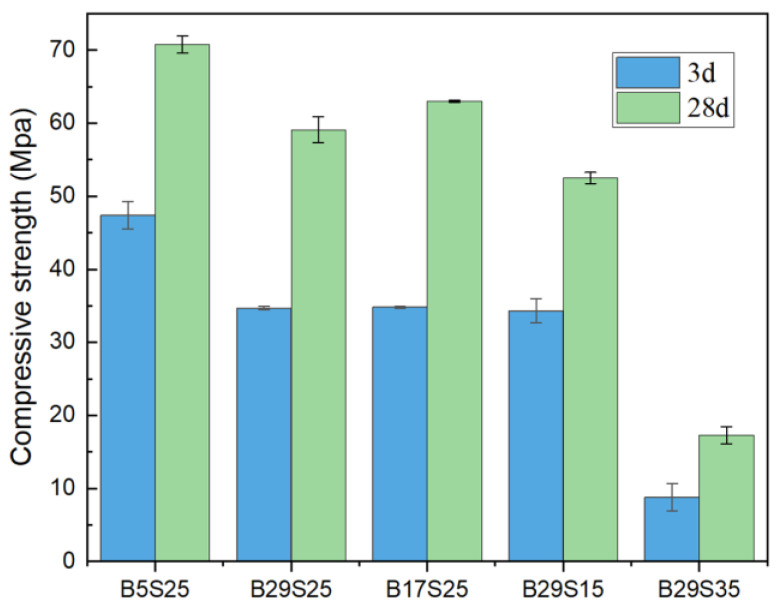
Compressive strength of MSWIBA GGBS composite cementitious materials activated by sodium silicate.

Figure 2 shows the compressive strength of the materials with different dosing ratios. The changes in compressive strength are evident. Before the reaction, the compressive strength at 3d is approximately half that at 28d. During the third reaction stage, a significant quantity of unreacted silicate mineral particles exists in the material, which means that with the increase in hydration reaction time, the degree of hydration reaction increases and compressive strength improves. In the experimental group B5S25, the 3d and 28d compressive strengths were 47.4 and 70.8 Mpa, respectively, with the highest compressive strengths. Compared with B29S25 and B17S25, with 17% and 29% MSWIBA, the 3d and 28d compressive strengths show decreasing tendencies with the increase in MSWIBA and the 3d compressive strengths reduce by 26.6% and 26.8%, respectively. The 28d compressive strengths decrease by 11.0% and 16.5%. It shows that with the increase in MSWIBA, many MSWIBA particles in the early stage of hydration of the unreacted particles increased, while the porosity between the material decreased. Hydration decreases and the compressive strength decreases with the increase in MSWIBA.

#### 3.1.2. Mechanical Properties of Different Sodium Silicate Dosages

Figure 2 shows that the 3- and 28-day compressive strengths of 15% sodium silicate and the 29% MSWIBA experimental group B29S15 were 34.3 and 52.5 Mpa, respectively. The 3- and 28-day compressive strengths of B29S25 were the highest compared to B29S15 and B29S35, which increased by 1.2% and 12.6% compared to B29S15. The compressive strengths of the B29S35 experimental group were the lowest, only 8.8 and 17.3 Mpa, i.e., 74.3% and 67.0% lower than B29S25, respectively. This means that while sodium silicate increased from 15% to 25%, the alkaline material also increased, enhancing the hydration of sodium silicate particles in the alkaline environment. Consequently, the degree of hydration reaction increased, the porosity decreased and promoted the generation of hardened slurry with strength, and the compressive strength increased. When the sodium silicate increased from 25% to 35%, the alkaline material became excessive, resulting in over-alkalinity. This inhibited the hydration of particles, causing a decrease in the degree of hydration reaction and the compressive strength.

### 3.2. Hydration Process

#### 3.2.1. Cumulative Heat

**Figure 3:**
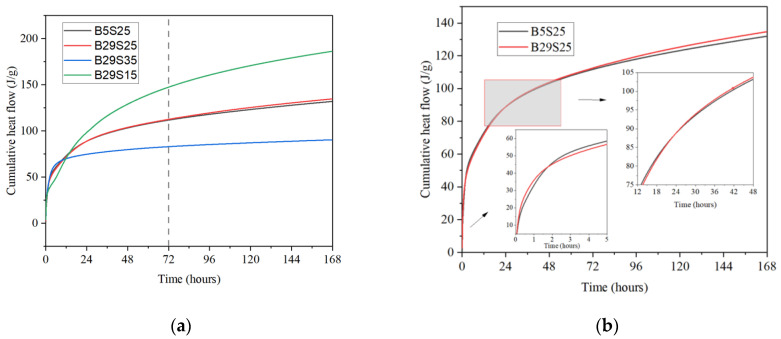
(**a**) Cumulative heat diagrams; (**b**) Cumulative heat for B5S25 and B29S25.

Figure 3a shows the cumulative exothermic curves of the four groups of materials with different ratios, from which it can be seen that the cumulative exothermic hydration time of each group increased with the increase in hydration reaction time. Additionally, with exothermic hydration, the mechanical compressive strength of the material also increased. The amount of exothermic hydration for B29S35 was significantly lower than that of the other experimental groups, as was its mechanical compressive strength. B29S15 exhibited the most significant amount of exothermic hydration and its mechanical compressive strength performed better, much higher than that of B29S35 and lower than that of the other experimental groups. The differences between B29S15 and B5S25 were minor, as shown in Figure 3b. Comparisons can be made that show that, approximately 1.5 h before and 24 h after the reaction, the exothermic hydration of B29S15 was slightly higher than that of B5S25, whereas in between, the exothermic hydration of B5S25 was somewhat higher. The amount of exothermic hydration was marginally higher.

#### 3.2.2. Heat Flow

**Figure 4:**
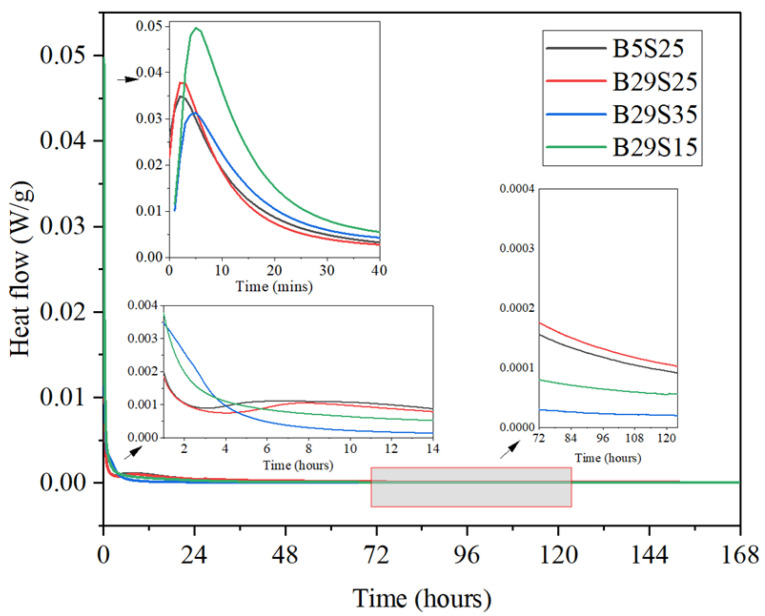
Heat flow diagrams of MSWIBA GGBS composite cementitious material activated by sodium silicate.

Figure 4 shows the calorimetric response and the exothermic process measured using isothermal calorimeter tests, summarized as follows.

The hydration process was roughly divided into five phases. An initial induction phase, an induction phase, an acceleration phase, a deceleration phase, and a steady state phase [42]. The materials did not undergo an apparent initial induction or have an induction period and there was a prominent exothermic peak at the beginning of hydration.

The first stage is the initial induction period; this initial stage is a transient state in which the mixed GGBS dissolves rapidly. At this time, the aluminum silicate precursor gradually dissolves and is destroyed, forming an unstable iso-phase [42,43].

The second phase is the induction phase, which proceeds from the initial induction phase. At this point, the Ca^2+^ in the cementitious materials dissolves with the alkali activator, causing the ionic concentration mixing solution to become too high and the dissolution of the aluminosilicates to be inhibited [42].

The third phase, the accelerated phase, ranges from 10 min to 2–3 h; at this time, with the dissolution of the alkali activator, the concentration of OH- in the solution increases and the activity of MSWIBA and GGBS is activated, under which hydration and polymerization reactions take place to produce gel products such as (N)C-A-S-H.

The fourth phase is deceleration, ranging from 2~3 h to 24 h; at this time, the hydration rate starts to slow down with the increase in hydration products because the surface of MSWIBA and GGBS particles is gradually covered by the hydration products, which reduces the contact area [42].

Phase 5 is the steady state stage, which occurs beyond 24 h. The hydration rate is relatively low and stable, which is called the stabilization period. At this time, the cementitious material gradually hardens from the paste, the hydration reaction forms a hardened slurry, and the pore structure becomes stable [42].

#### 3.2.3. Hydration Exothermic Equivalent Age Modeling

The heat of hydration was expressed using hyperbolic form [22], as shown in the following Equation (1):

(1) $$Q\left(t\right)=Q_{\max}t/\left(n+t\right)$$

where *n* is the heat of hydration at half of the age. The above equation can be rewritten in the following form, as shown in the following Equation (2):

(2) $$\frac{t}{Q\left(t\right)}=\frac{n}{Q_{\max}}+\frac{t}{Q_{\max}}$$

First, the ratio *t*/*Q*(*t*) was chosen as the vertical coordinate and *t* as the horizontal coordinate for a graph. Then, a fitting analysis was performed to obtain the slope of the line as 1/*Qmax* and the line intercept in the vertical coordinate as *n*/*Qmax*, leading to the value of *n*. This critical parameter helped us accurately describe the hydraulic exothermic process.

**Figure 5:**
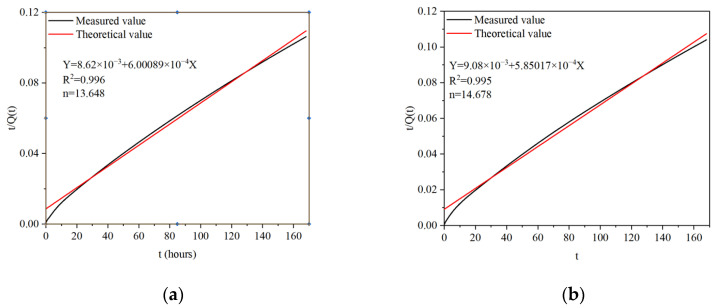
(**a**) Model parameter-solving diagram for B5S25; (**b**) model parameter-solving diagram for B29S25.

The equations can be obtained by calculation using *Qmax*. Figure 5 shows the hydration and heat release models of B5S25 and B29S25, as shown in Equations (3) and (4), respectively.

(3) $$Q(t)=\frac{10,000t}{86.2+6.00089t}$$

(4) $$Q(t)=\frac{10,000t}{90.8+5.85017t}$$

**Figure 6:**
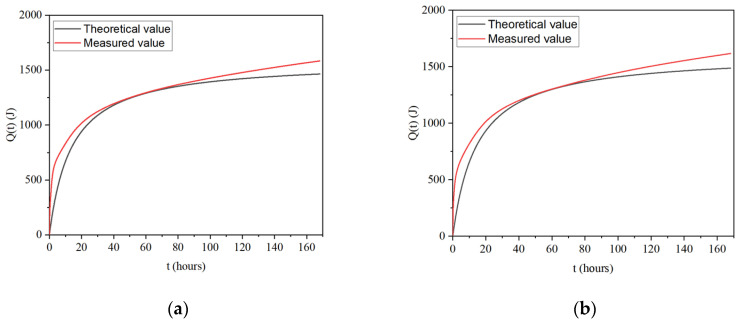
(**a**) Comparison between the theoretical and measured values for B5S25; (**b**) comparison between the theoretical and measured values for B29S25.

Figure 6 illustrates the cumulative heat release of the material, as predicted by the hydration heat release model, alongside the accumulated heat release measured during testing. It can be seen from the figure that with an increase in the MSWIBA content, the total amount of accumulated heat release decreased and the theoretical value became less than the test value during the heat release. The difference did not change much in the 60 h before the heat release of hydration. After 72 h, the difference increased with time and the degree fitting degree worsened in the late stage of hydration heat release.

### 3.3. Hydration Products and Properties

#### 3.3.1. XRD Analysis

**Figure 7:**
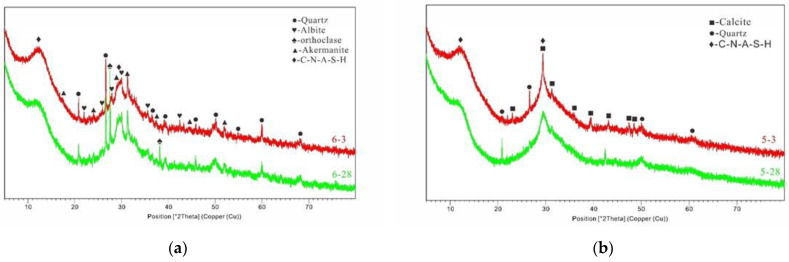
(**a**) XRD posotion patterns of B5S25; (**b**) XRD posotion patterns of B29S25.

Figure 7a,b compares the ratios of the three materials after 3 and 28 days of curing B5S25 and B29S25. Compared to the XRD test curves of the raw materials, the X-ray diffraction peaks of the alkali-activated MSWIBA were relatively small in angle, indicating the formation of a new amorphous gel [44]. The broader diffraction peaks were mainly associated with the amorphous material of the feedstock and the newly formed amorphous gels of the reaction products. Using XRD analysis, it is difficult to identify and differentiate amorphous N-A-S-H and C-A-S-H gels. The quartz and mullite crystals in the MSWIBA were not fully involved in the reaction during the hydration process. They demonstrated distinct diffraction peaks in the XRD patterns.

In contrast, the difference between specimen peaks was relatively small at three days, indicating that adding MSWIBA weakened the early hydration of the alkali-stimulated composite collodion materials [44]. In addition, the trend of high to low is consistent with the results of the mechanical properties of the slurry. A comparison of the plots of specimen B5S25 showed that the amount of hydration products increased with the length of hydration [44]. The XRD plots of group B29S25 showed changes in the physical phases after 3 and 28 days of curing. The MSWIBA content increased from 5% to 29% and the physical phases of the main hydration products in the gelling system were similar. However, an increase in the diffraction peaks of the new and firmer phases was found.

In conclusion, the physical phases of quartz and calcite were observed in the XRD patterns of the two sets of diagrams. As the hydration process proceeded, the quartz and calcite contents decreased, suggesting that these two substances transformed into different substances during the hydration process. The reduction in quartz and calcite indicates that these two substances are involved in the hydration reaction and the primary substances of the hydration reaction are similar. The C-A-S-H peaks of the 28-day groups were higher than the corresponding positions of the 7-day peaks, which indicates that the degree of hydration reaction was higher.

#### 3.3.2. Fourier-Transform Infrared (FTIR) Analysis

**Figure 8:**
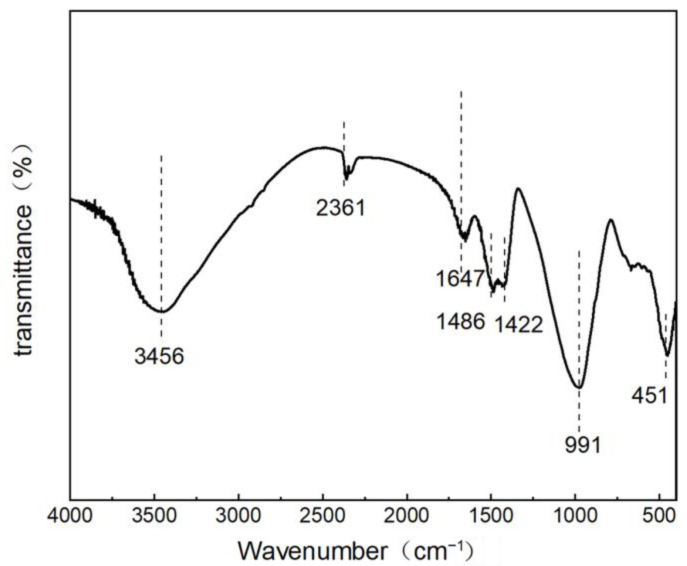
FTIR spectrum of B5S25.

FTIR spectroscopy was used to obtain the structural characteristics. Figure 8 shows the structure of the functional groups of B5S25, in which an O-H telescopic vibrational absorption peak at 3464 cm^−1^ can be observed, generated by the telescopic vibration of H-O-H in water [31]. There was also an O-H bending vibration peak at 1651 cm^−1^, generated by the bending vibration of O-H in water. These peaks indicate the amount of chemically bound water in the alkali-activated MSWIBA GGBS binder. The higher intensity of the peaks indicates the presence of more chemically bound water in the binder [43]. Moreover, no distinct or sharp bands associated with the stretching vibration of the OH single bond in Ca(OH)_2_ were observed at 3644 cm^−1^, whereas the presence of the absorption band material of the Al(Mg)Si-OH phase was seen in the infrared spectral map of the MSWIBA at 3620 cm^−1^. This indicates that the aluminosilicate in MSWIBA actively participates in the hydration reaction process during alkali activation, thus eliminating the vibrations of Al(Mg)Si-OH [31,45]. This suggests that the alkaline chemical environment plays a crucial role in reducing the crystallinity of quartz in MSWIBA and domestic waste incineration GGBS.

The absorption peak at 1420 cm^−1^ resulted from the C-C stretching vibration caused by the symmetric stretching vibration of CO_3_^2−^ at 3464 cm^−1^, which accompanied a broad water peak with low intensity. This indicates that water occupied a certain proportion of the sample, suggesting that the binder contained a higher amount of chemically bound water. In the range of 600 to 1200 cm^−1^, vibrational peaks with Si-O-Ti (T = Si and Al) were observed, indicating that an aluminosilicate network structure existed in all of the hydration products. This suggests that the aluminosilicates in the bottom GGBS of domestic waste incineration actively participate in the hydration reaction process during the alkali activation of the production process of bottom GGBS in domestic waste incineration. The results show that the samples were present in the C-A-S-H and calcium alumina, which is consistent with the X-ray diffraction and SEM-EDS analysis results.

#### 3.3.3. Scanning Electron Microscopy

According to previous results, amorphous gels usually consist of C-A-S-H and N-A-S-H gels coexisting in alkali-activated GGBS/systems and the analyses showed the presence of N-A-S-H gels, C-(A)-S-H gels, and some unreacted MSWIBA and GGBS particles. These components showed a disordered lattice structure and excellent compatibility [31,46,47], indicating that sodium silicate’s content significantly influenced the surface morphology and structure of the composite gelling material.

**Figure 9:**
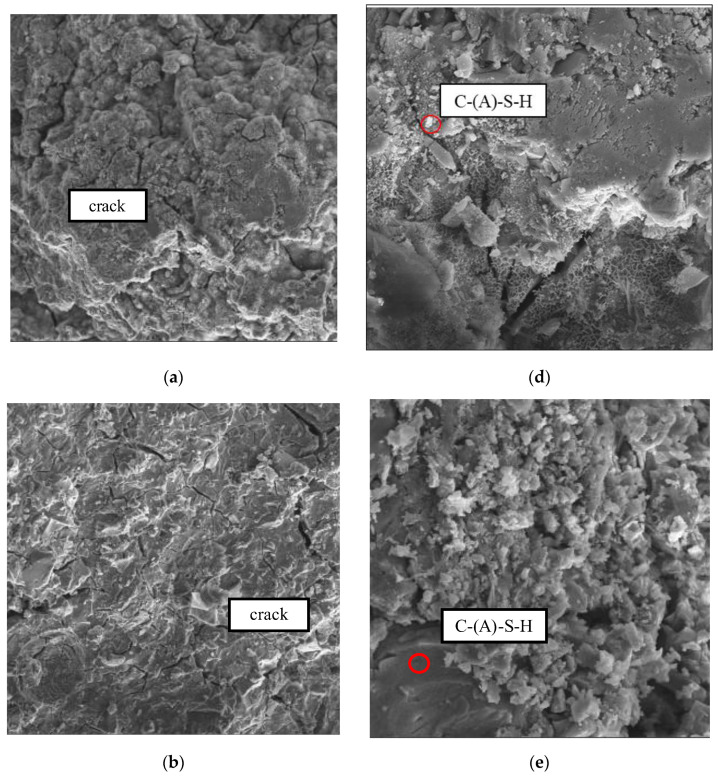

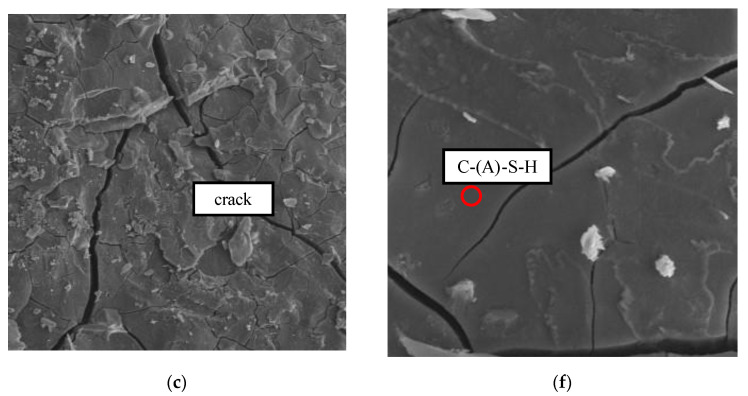
(**a**,**d**) SEM observation of B29S15 at 28 days; (**b**,**e**) SEM observation of B29S35 at 28 days; (**c**,**f**) SEM observation of B29S25 at 28 days.

As can be seen from Figure 9a–c, the samples in groups B29S15, B29S35, and B29S25 have multiple microcracks due to drying shrinkage. Among them, the width and number of microcracks on the surface of B29S35 were significantly higher than that of B29S15 and there were few microcracks in B29S25. This is because when the alkali exciter dosage is 15%, it cannot provide enough alkaline material. Consequently, the activity of the gelling system is insufficient and the particles cannot react sufficiently, resulting in lower strength and a loose micro-morphology, indicating the presence of many unhydrated particles. When the alkali exciter content was increased to 25%, the activity of the gelling system increased, thus promoting the hydration reaction effect, allowing the particles to become fully dissolved and hydrated. As per the figure, the micrograph of the B29S25 specimen shows dense characteristics, with the hydration reaction products tightly wrapped around the unreacted substrate particles. At the same time, the substrate is well compacted and no apparent microcracks were found, resulting in excellent performance in terms of the compressive properties of the hardened slurry. When the alkali exciter dosage exceeded 25% to 35%, it was in excess, inhibiting the occurrence of the hydration reaction. Therefore, a large number of particles were exposed and they were not sufficiently hydrated to generate a crystalline phase. The loose morphology of B29S35 with a vast number of microcracks can be seen in Figure 9b.

The dense amorphous gel phase C-(A)-S-H with large-size silicate particles can be seen in Figure 9d–f, in which B29S35 had the most and B29S35 the least. In Figure 9e, a network-like gel phase can be seen and the 35% alkali stimulant dosing of B29S35 contained a large number of unhydrated particles compared to B29S25, which also had partially exposed and unhydrated particles. The 28-day compressive strength of the B29S35 sample was only approximately one-third of that of B29S25. Many needle- and rod-like AFt crystals can be seen in Figure 9f, enhancing the interconnection with the C-A-S-H gel.

The results of this study show that the performance of the micro-morphological characteristics and compressive strength test results is consistent with the alkalinity of the alkali exciter, being one of the very important factors in the formation of MSWIBA composite cementitious materials and the final hydration products. With an increase in alkali exciter dosage from 15% to 25%, the increase in alkali exciters significantly promoted the conversion of free alkali to bonded alkali and the stimulation of the activator was enhanced, which helped the hydration reaction occur. A more hydrated crystalline phase was generated and the strength of the cementitious system improved. When the alkali exciter dosage increased from 25% to 35%, the alkali exciter dosage became excessive, resulting in an over-alkali effect, reducing the stimulation ability of the activator, and inhibiting the hydration reaction. Additionally, there were a large number of unhydrated particles and the microscopic morphology showed that the product was very loose, with a large number of microcracks, resulting in a significant decrease in compressive strength.

## 4. Summary

This research investigated the preparation and hydration properties of the sodium silicate activation of MSWIBA–GGBS composite cementitious materials, leading to the following conclusions.

The compressive strength decreased with an increase in MSWIBA. When the sodium silicate content was below 25%, the compressive strength exhibited an increasing trend with higher silicate levels. However, when the content was above 25%, the compressive strength decreased as the silicate level increased.

The hydration process can be divided into five phases. The heat is reduced with the increase in MSWIBA and the hyperbolic form is modeled as less than the test value.

As the hydration process proceeded, the quartz and calcite contents decreased, and activated aluminum participated in the hydration reaction. The XRD, FTIR, and SEM results showed that there was an aluminosilicate network structure in the hydration products, mainly N-A-S-H and C-A-S-H gels.

## Figures and Tables

**Table 1:** The main chemical compositions of MSWIBA and GGBS (wt%).

Oxides	SiO_2_	CaO	Al_2_O_3_	Fe_2_O_3_	P_2_O_5_	Na_2_O	MgO	SO_3_	K_2_O
MSWIBA	37.79	28.60	9.34	5.94	5.22	3.25	2.74	2.33	1.52
GGBS	30.49	40.83	15.26	0.32	0.02	0.49	8.50	2.08	0.45

**Table 2:** The main chemical compositions of MSWIBA and GGBS (wt%).

Number	MSWIBA	Sodium Silicate	GGBS	W/C
B5S25	5	25	70	0.5
B29S25	29	25	46	0.5
B17S25	17	25	58	0.5
B29S15	29	15	56	0.5
B29S35	29	35	36	0.5

## Data Availability

Data are contained within the article.

## References

[1] Silva R.V., de Brito J., Lynn C.J., Dhir R.K. (2019). Environmental impacts of the use of bottom ashes from municipal solid waste incineration: A review. Resour. Conserv. Recycl..

[2] Barros R.M., Siddique R., Belarbi R. (2022). 4-Municipal solid waste ash. Sustainable Concrete Made with Ashes and Dust from Different Sources.

[3] Stegemann J.A., Schneider J., Baetz B.W., Murphy K.L. (1995). Lysimeter washing of MSW incinerator bottom ash. Waste Manag. Res..

[4] Liu J., Wang Z., Li Z., Xie G., Zhang W., Jin H., Xing F. (2024). Investigation of the cyclic separation of dioxins from municipal solid waste incineration fly ash by using fat. J. Clean. Prod..

[5] Joseph A.M., Snellings R., Van den Heede P., Matthys S., De Belie N. (2018). The Use of Municipal Solid Waste Incineration Ash in Various Building Materials: A Belgian Point of View. Materials.

[6] You Z., Mills-Beale J., Yang X., Dai Q. (2012). Alternative Materials for Sustainable Transportation.

[7] Provis J.L. (2014). Geopolymers and other alkali activated materials: Why, how, and what?. Mater. Struct..

[8] Ali M.B., Saidur R., Hossain M.S. (2011). A review on emission analysis in cement industries. Renew. Sustain. Energy Rev..

[9] Wang J., Zhai Z., Jing Y., Zhang C. (2011). Influence analysis of building types and climate zones on energetic, economic and environmental performances of BCHP systems. Appl. Energy.

[10] González-Torres M., Pérez-Lombard L., Coronel J.F., Maestre I.R., Yan D. (2022). A review on buildings energy information: Trends, end-uses, fuels and drivers. Energy Rep..

[11] Alves B.I., Marvila M.T., Linhares Júnior J.A., Vieira C.M., Alexandre J., de Azevedo A.R. (2024). Alkaline Activation of Binders: A Comparative Study. Materials.

[12] Gao X., Yuan B., Yu Q.L., Brouwers H.J.H. (2017). Characterization and application of municipal solid waste incineration (MSWI) bottom ash and waste granite powder in alkali activated slag. J. Clean. Prod..

[13] Tang P., Chen W., Xuan D., Zuo Y., Poon C.S. (2020). Investigation of cementitious properties of different constituents in municipal solid waste incineration bottom ash as supplementary cementitious materials. J. Clean. Prod..

[14] Yamaguchi N., Nagaishi M., Kisu K., Nakamura Y., Ikeda K. (2013). Preparation of monolithic geopolymer materials from urban waste incineration slags. J.-Ceram. Soc. Jpn..

[15] Łach M., Mierzwiński D., Korniejenko K., Mikuła J., Hebda M. (2018). Geopolymers as a material suitable for immobilization of fly ash from municipal waste incineration plants. J. Air Waste Manag. Assoc..

[16] Peceño B., Luna-Galiano Y., Varela F., Alonso-Fariñas B., Leiva C. (2024). Study of a Fire-Resistant Plate Containing Fly Ashes Generated from Municipal Waste Incinerator: Fire and Mechanical Characteristics and Environmental Life Cycle Assessment. Materials.

[17] Vilarinho I.S., Guimarães G., Labrincha J.A., Seabra M.P. (2023). Development of Eco-Mortars with the Incorporation of Municipal Solid Wastes Incineration Ash. Materials.

[18] Malaiškienė J., Spudulis E., Stonys R. (2023). The Effect of Milled Municipal Solid Waste Incineration Bottom Ash on Cement Hydration and Mortar Properties. Materials.

[19] Thomas M., Ślosarczyk A. (2023). Effect of Municipal Solid Waste Slag on the Durability of Cementitious Composites in Terms of Resistance to Freeze–Thaw Cycling. Materials.

[20] Poranek N., Łaźniewska-Piekarczyk B., Lombardi L., Czajkowski A., Bogacka M., Pikoń K. (2022). Green Deal and Circular Economy of Bottom Ash Waste Management in Building Industry—Alkali (NaOH) Pre-Treatment. Materials.

[21] Czop M., Łaźniewska-Piekarczyk B. (2020). Use of Slag from the Combustion of Solid Municipal Waste as a Partial Replacement of Cement in Mortar and Concrete. Materials.

[22] Lin K.L., Lin D.F. (2006). Hydration characteristics of municipal solid waste incinerator bottom ash slag as a pozzolanic material for use in cement. Cem. Concr. Compos..

[23] Lynn C.J., Dhir R.K., Ghataora G.S. (2016). Municipal incinerated bottom ash characteristics and potential for use as aggregate in concrete. Constr. Build. Mater..

[24] Pouhet R., Cyr M. (2016). Carbonation in the pore solution of metakaolin-based geopolymer. Cem. Concr. Res..

[25] Ryu G.S., Lee Y.B., Koh K.T., Chung Y.S. (2013). The mechanical properties of fly ash-based geopolymer concrete with alkaline activators. Constr. Build. Mater..

[26] Huang G., Ji Y., Li J., Hou Z., Jin C. (2018). Use of slaked lime and Portland cement to improve the resistance of MSWI bottom ash-GBFS geopolymer concrete against carbonation. Constr. Build. Mater..

[27] Xiaolong Z., Shiyu Z., Hui L., Yingliang Z. (2021). Disposal of mine tailings via geopolymerization. J. Clean. Prod..

[28] Krivenko P., Pushkareva E., Chirkova V. (1985). Processes of the calcium silicate hydration in the presence of alkali-metal compounds. Izv. Vyss. Uchebnykh Zaved. Khimiya I Khimicheskaya Tekhnologiya.

[29] Provis J.L., Duxson P., Van Deventer J.S.J. (2007). Geopolymer Technology and the Search for a Low-CO_2_ Alternative to Concrete.

[30] Davidovits J. (1989). Geopolymers and geopolymeric materials. J. Therm. Anal..

[31] Moussadik A., Ouzoun F., Ez-zaki H., Saadi M., Diouri A. (2023). Mineralogical study of a binder based on alkali-activated coal gangue. Mater. Today Proc..

[32] Setlak K., Mikuła J., Łach M. (2023). Application of Industrial Waste Materials by Alkaline Activation for Use as Geopolymer Binders. Materials.

[33] Kwek S.Y., Awang H., Cheah C.B.J.M. (2021). Influence of liquid-to-solid and alkaline activator (Sodium silicate to sodium hydroxide) ratios on fresh and hardened properties of alkali-activated palm oil fuel ash geopolymer. Materials.

[34] Sun K., Peng X., Wang S., Zeng L., Ran P., Ji G.J.C., Materials B. (2020). Effect of nano-SiO_2_ on the efflorescence of an alkali-activated metakaolin mortar. Constr. Build. Mater..

[35] Adeleke B.O., Kinuthia J.M., Oti J., Pirrie D., Power M. (2024). Mechanical and Microstructural Investigation of Geopolymer Concrete Incorporating Recycled Waste Plastic Aggregate. Materials.

[36] Sirotti M., Delsaute B., Staquet S. (2023). New Experimental Evidence for Drying Shrinkage of Alkali-Activated Slag with Sodium Hydroxide. Materials.

[37] Naqi A., Delsaute B., Königsberger M., Staquet S. (2023). Effect of Solution-to-Binder Ratio and Alkalinity on Setting and Early-Age Properties of Alkali-Activated Slag-Fly Ash Binders. Materials.

[38] (2008). Granulated Blastfurnace Slag Used for Cement Production.

[39] (2017). Ground Granulated Blast Furnace Slag Used for Cement, Mortar and Concrete.

[40] (2021). Test Method of Cement Mortar Strength (ISO Method).

[41] Asadizadeh M., Hedayat A., Tunstall L., Gonzalez J.A.V., Alvarado J.W.V., Neira M.T. (2024). The impact of slag on the process of geopolymerization and the mechanical performance of mine-tailings-based alkali-activated lightweight aggregates. Constr. Build. Mater..

[42] Zhao J., Long B., Yang G., Cheng Z., Liu Q. (2022). Characteristics of alkali-activated slag powder mixing with seawater: Workability, hydration reaction kinetics and mechanism. Case Stud. Constr. Mater..

[43] Zhang X., Wang W., Zhang Y., Gu X. (2024). Research on hydration characteristics of OSR-GGBFS-FA alkali-activated materials. Constr. Build. Mater..

[44] Huang G., Yang K., Sun Y., Lu Z., Zhang X., Zuo L., Feng Y., Qian R., Qi Y., Ji Y. (2020). Influence of NaOH content on the alkali conversion mechanism in MSWI bottom ash alkali-activated mortars. Constr. Build. Mater..

[45] Mitrović A., Zdujić M. (2014). Preparation of pozzolanic addition by mechanical treatment of kaolin clay. Int. J. Miner. Process..

[46] Li B., Liu Z., Sun Q., Yang L. (2024). Preparation of alkali-activated nickel slag-based cemented tailings backfill: Workability, strength characteristics, localized deformation and hydration mechanism. Constr. Build. Mater..

[47] Ahmad M.R., Qian L.-P., Fang Y., Wang A., Dai J.-G. (2023). A multiscale study on gel composition of hybrid alkali-activated materials partially utilizing air pollution control residue as an activator. Cem. Concr. Compos..

